# Interactively learning behavior trees from imperfect human demonstrations

**DOI:** 10.3389/frobt.2023.1152595

**Published:** 2023-07-12

**Authors:** Lisa Scherf, Aljoscha Schmidt, Suman Pal, Dorothea Koert

**Affiliations:** ^1^ Interactive AI & Cognitive Models for Human-AI Interaction (IKIDA), Technische Universität Darmstadt, Darmstadt, Germany; ^2^ Centre of Cognitive Science, Technische Universität Darmstadt, Darmstadt, Germany; ^3^ Telekinesis, Intelligent Autonomous Systems Group, Department of Computer Science, Technische Universität Darmstadt, Darmstadt, Germany

**Keywords:** human-robot interaction, interactive task learning, behavior trees, learning from demonstration, robotic tasks, user studies, failure detection, failure recovery

## Abstract

**Introduction:** In Interactive Task Learning (ITL), an agent learns a new task through natural interaction with a human instructor. Behavior Trees (BTs) offer a reactive, modular, and interpretable way of encoding task descriptions but have not yet been applied a lot in robotic ITL settings. Most existing approaches that learn a BT from human demonstrations require the user to specify each action step-by-step or do not allow for adapting a learned BT without the need to repeat the entire teaching process from scratch.

**Method:** We propose a new framework to directly learn a BT from only a few human task demonstrations recorded as RGB-D video streams. We automatically extract continuous pre- and post-conditions for BT action nodes from visual features and use a Backchaining approach to build a reactive BT. In a user study on how non-experts provide and vary demonstrations, we identify three common failure cases of an BT learned from potentially imperfect initial human demonstrations. We offer a way to interactively resolve these failure cases by refining the existing BT through interaction with a user over a web-interface. Specifically, failure cases or unknown states are detected automatically during the execution of a learned BT and the initial BT is adjusted or extended according to the provided user input.

**Evaluation and results:** We evaluate our approach on a robotic trash disposal task with 20 human participants and demonstrate that our method is capable of learning reactive BTs from only a few human demonstrations and interactively resolving possible failure cases at runtime.

## 1 Introduction

The multitude of possible tasks and user preferences in everyday scenarios renders pure pre-programming of future robots inadequate. The ability to learn new tasks from non-expert users becomes therefore a key component for the development of intelligent robotic systems (Laird et al., 2017).

Behavior Trees (BTs) offer a reactive, modular, and interpretable way of encoding task descriptions and have recently gained increasing attention in the robotic community (Marzinotto et al., 2014; Paxton et al., 2017; Colledanchise and Ögren, 2018; Fusaro et al., 2021).

However, only a few existing approaches learn BTs directly from human task demonstrations (Robertson and Watson, 2015; Sagredo-Olivenza et al., 2017;French et al., 2019; Gustavsson et al., 2021) or allow for adapting or refining the learned BT without having to repeat the entire teaching process (Helenon et al., 2021; Iovino et al., 2022a). In particular, when dealing with incomplete or imperfect task demonstrations this results in frustrating teaching routines and a higher risk of failures at execution time.

In this paper, we propose ILBERT (Interactively Learning BEhavioR Trees), a new framework for learning a BT from only a few human demonstrations and interactively refine the learned BT during runtime. We use visual feature extraction for high-level action segmentation and a backchaining approach to learn an initial BT directly from video demonstrations. At execution time, we resolve failure cases by refining or extending the learned BT according to interactive user input over a graphical user interface.

**FIGURE 1:**
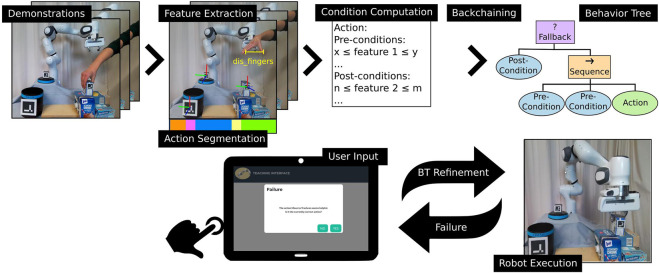
Overview of the proposed approach. First, the user provides few demonstrations of a task. Task-relevant features are extracted and the demonstrations are segmented into high-level action sequences. Based on the features and action labels, pre- and post-conditions for all actions are computed. A Backchaining approach is used to build an initial Behavior Tree from those conditions which can directly be executed by the robot. During execution, failure cases are automatically detected and resolved using input provided by the user via a web-interface and the initial Behavior Tree and corresponding action conditions are thereby iteratively adapted.

To determine different states for action execution in the BT, we extract pre- and post-conditions from the human task demonstrations. Unlike related approaches (Colledanchise et al., 2019; Gustavsson et al., 2021; Iovino et al., 2021), we use continuous conditions instead of binary ones and extract these conditions from human video demonstrations instead of manually pre-defining them for each action. However, the initial demonstrations and resulting pre- and post-conditions may not cover all situations that can occur at execution time of the learned BT. Therefore, during task execution, our approach automatically detects states not seen during demonstrations and requests additional input from the user to refine or extend the initially learned BT. Figure 1 summarizes our proposed approach.

We investigate two main research questions in experimental evaluations on a robotic trash disposal task with a Franka Erika Panda robot. First, we analyze human demonstrations in a pilot study in order to evaluate what kind of task demonstrations non-expert users provide to our robot and when and why BTs, learned with our proposed approach from this initial set of demonstrations fail. Second, we propose an interactive approach to refine and extend the learned BT at runtime to resolve the observed failure cases and evaluate our approach in a subsequent user study. Demonstration data from the pilot study is used to train an action classifier that predicts high-level action sequences from extracted video features for the second study. The experimental evaluation shows that our proposed approach results in successful refinement and solving of potential failure cases after initial BT learning. In addition, we analyze user satisfaction regarding the resulting task performance and interaction with the overall system.

Overall, the main contributions of our paper are the following. First, we present an approach to directly learn a BT from human video demonstration, including automatic action segmentation and extraction of pre- and post-conditions for action execution using visual features. Second, we analyze potential cases where imperfect non-expert human demonstrations may lead to failure cases of the initially generated BT. Third, we implement and evaluate an interactive approach to resolve such failure cases during execution by refining or extending the BT with user input over a graphical user interface and additional demonstrations.

The rest of the paper is structured as follows. In Section 2, we provide a summary on BTs and discuss related approaches. Afterwards Section 3 introduces our novel framework for interactive learning of a BT from a few human video demonstrations. In Section 4, we analyze potential failure cases resulting from imperfect human demonstrations, evaluate our proposed method on a robotic task with human users, and discuss the results. Lastly, Section 5 concludes the paper and gives an outlook on future research directions.

## 2 Background and related work

In this section, we first provide a summary on the concept of Behavior Trees and afterwards discuss related works on learning Behavior Trees.

### 2.1 Behavior trees

Behavior Trees are control structures used to switch between different tasks in an autonomous agent. They initially emerged in the gaming industry as an alternative to Finite State Machines (Mateas and Stern, 2002; Millington and Funge, 2018). Over the last years, they have shown their great potential for structuring robot behaviors (Colledanchise and Ögren, 2018; Iovino et al., 2022b).

In comparison to Finite State Machines, BTs provide the advantages of uncoupled modularity and more straightforward reusability of (sub-)behaviors as well as built-in reactiveness and improved human readability (Colledanchise and Ögren, 2016; Colledanchise and Ögren, 2018; Han et al., 2021).

A BT is a directed rooted tree built from internal nodes and leaf nodes. The leaf nodes execute (sub-)tasks, i.e., behaviors, whereas all internal nodes are control flow nodes. An example BT can be seen on the top right in Figure 1, where control nodes are depicted in rectangular shapes and execution nodes as ellipsoids. During the execution of the BT, the root node is *ticked* at a specified frequency and passes the tick signal to its children. A ticked node returns *RUNNING* to its parent during execution, *SUCCESS* if its goal is achieved, or *FAILURE* otherwise. Execution nodes can encode Actions for a robot to execute or conditions that may encode, e.g., checks of environmental or internal status or sensor feedback. The most commonly used control nodes are Sequence nodes and Fallback nodes (depicted in orange with symbol → and purple with symbol ? in Figure 1). Sequence nodes execute the subsequent children nodes in a sequence and return *SUCCESS* if all children succeeded or *FAILURE* once one of the children fails. Fallback nodes also execute their children in a sequence but return *FAILURE* only if all children fail and *SUCCESS* as soon as one child succeeds.

It can be noted that BTs are, by definition, close to Decision Trees (Colledanchise and Ögren, 2016; French et al., 2019). However, BTs offer built-in reactivity since nodes can be executed for longer than one tick using the *RUNNING* state, which allows other actions to preempt running ones by returning *FAILURE*.

There are different existing code frameworks that implement BTs (Ghzouli et al., 2020). For the experiment in this paper, we used the BehaviorTree.CPP Library (Faconti, 2018) due to its compatibility with ROS. Trees are defined here using an XML-based format and can be visualized using the graphical user interface *Groot* (Faconti, 2018, Faconti, 2019).

For further details on BTs in robotics and AI, we refer to (Colledanchise and Ögren, 2018).

### 2.2 Learning behavior trees

There are several approaches to design BTs for specific tasks (Iovino et al., 2022b). BTs can be hand-coded or constructed manually using supporting design tools, such as the GUI Editor Groot. CoSTAR (Paxton et al., 2017) enables non-expert users to create robust robotic task plans using a BT-based task editor integrating perception. This simplifies implementation but manually designing the tree step-by-step is still necessary and challenging for more complex tasks. BTs have also been used as control structures to manually combine movement primitives over a graphical user interface in guided robot skill learning (Knaust and Koert, 2021). Besides manual construction, BTs can be built using a planning algorithm to compute a plan to solve a task and then convert this plan into a BT. Genetic Programming can, for example, be used to automatically build a BT starting from a set of actions and conditions and a reward or fitness function (Scheper et al., 2016; Colledanchise et al., 2018; Iovino et al., 2021). However, defining such a function can be difficult, especially for everyday-life users. Similarly, Banerjee (2018) proposes to first autonomously learn a reinforcement learning control policy and then convert this policy into a BT based on canonical BTs, which is a reduced representation of BTs.

Learning from demonstration (LfD) offers a promising alternative that particularly also enables non-expert users to teach robots new tasks (Ravichandar et al., 2020). However, to the best of our knowledge, there are so far only few works that have learned behavior trees from demonstrations (Robertson and Watson, 2015; Sagredo-Olivenza et al., 2017; French et al., 2019; Gustavsson et al., 2021).

One such approach is to learn a decision tree (DT) from demonstrated state-action pairs and afterwards convert the learned DT into an equivalent Behavior Tree (Sagredo-Olivenza et al., 2017; French et al., 2019). This was proposed first to assist game designers in programming non-player characters (Sagredo-Olivenza et al., 2017) and later extended and applied to learn a robot house cleaning task (French et al., 2019). Specifically, French et al. (2019) generate a Decision Tree (DT) from user demonstrations in the form of selected actions via a user interface.

Gustavsson et al. (2021) propose a method to learn a BT from kinesthetic demonstrations. In addition, they propose a clustering approach to identify adequate reference frames for each action. The BT is built using Backchaining with pre-defined binary pre- and post-conditions. The Backchaining algorithm was first proposed by Colledanchise et al. (2019) and presents a planner to automatically grow a BT. The algorithm grows the tree iteratively by replacing failing pre-conditions with subtrees representing an action with an appropriate post-condition that satisfies the failed condition. Styrud et al. (2022) combine Genetic Programming with Backchaining to counterbalance the shortcomings of both methods and make learning more efficient. Our method also uses Backchaining to build a BT. However, we automatically extract pre- and post-conditions for each action from human demonstrations instead of defining them manually beforehand as opposed to the approaches in Gustavsson et al. (2021); Colledanchise et al. (2019). In addition, we use continuous pre- and post-conditions instead of binary features (Colledanchise et al., 2019; Gustavsson et al., 2021).

An alternative approach to learn a BT from human demonstrations is to directly map all demonstrations to subtrees consisting of Sequence nodes of all shown actions and place the subtree under a Fallback node in the BT. Robertson and Watson (2015) apply this method to learn a BT for the strategy game StarCraft. However, this results in large and hard-to-read BTs (> 50.000 nodes) whose structure limits reactivity.

Few works interactively learn or refine BTs through interaction with a human user. Suddrey et al. (2022) build a BT based on natural language instructions and use an interactive dialogue with the user to request additional information and resolve ambiguities. Similarly, Iovino et al. (2022a) combine the method of Gustavsson et al. (2021) with an interactive disambiguation framework (Doğan et al., 2022) to resolve ambiguities in a scene during BT execution through verbal interaction with the user. However, they focus solely on failure cases that arise from ambiguous objects in the scene. In Helenon et al. (2021), speech commands are combined with gestures in order to learn a BT. The approach allows incrementally learning tasks with growing complexity by interacting with the user. However, the user has to specify each action step-by-step, which can be cumbersome for complex tasks. In contrast to most interactive approaches to learn a BT (Helenon et al., 2021; Doğan et al., 2022; Suddrey et al., 2022), we directly learn a BT from video recordings of full human task demonstrations.

Overall we found a lack of evaluations of the proposed systems for BT learning with non-expert users on robotic tasks (Helenon et al., 2021; Iovino et al., 2021) and a lack of user studies with a focus on physical demonstrations of complete task sequences (Colledanchise et al., 2018; Gustavsson et al., 2021; Suddrey et al., 2022).

## 3 Interactively learning behavior trees from demonstrations

This section introduces our novel framework for Interactively Learning BEhavioR Trees from a few human demonstrations (ILBERT). In contrast to related approaches, task demonstrations are directly recorded as RGB-D data. We automatically extract task-relevant features and segment the demonstrations into high-level action sequences (Section 3.1). Based on the features and action sequences, pre- and post-conditions for each action are learned (Section 3.2.1), and an initial BT is built using Backchaining (Colledanchise et al., 2019) (Section 3.2.2). This initial BT might not cover all possible situations because of the limited number of potentially imperfect demonstrations. Therefore, failure cases that might occur during the execution of the learned BT are detected automatically and can be interactively resolved through user input via a web-interface (Section 3.3). The initial BT and corresponding conditions are updated accordingly. Figure 1 shows an overview of the pipeline. In the following, we explain each step in more detail.

### 3.1 Feature extraction and action segmentation

In contrast to other related approaches (French et al., 2019; Helenon et al., 2021), we want to directly learn a BT from recordings of complete human task executions instead of requiring the user to explain each action step-by-step.

Therefore, we record human task demonstrations with a RGB-D camera and segment these recordings into high-level action sequences $a_{0}^{d},\ldots ,a_{N^{d}}^{d}$ for each demonstration d∈D using a pre-trained classifier that maps a sliding window of *i* frames over a set of *j* features $x_{0},\ldots x_{j}\in X$ to one of *m* actions *a*_0_, … *a*_*m*_ in a set of pre-defined actions A :

$$c_{\theta }\left(X_{0..i}\right):R^{i\times j}\mapsto A$$ (1)

where ***θ*** denotes the model parameters. Inspired by Sieb et al. (2020), we use object-object and hand-object distances as features X for action segmentation. In order to extract those features, we use MediaPipe (Zhang et al., 2020) to infer relevant 3D landmarks (i.e., wrist, thumb, and index-fingertip) of the user’s hand for each video frame. Using ArUco markers, we additionally obtain a 3D pose estimation of task-relevant objects (i.e., trash, trashcan, and lid). Since the ArUco detection fails to detect the marker during fast movements of the object due to motion blur, we are using the CMT tracking algorithm (Nebehay and Pflugfelder, 2015), in addition. It is a keypoint-based method for long-term model-free object tracking. The tracker is re-initialized each time the ArUco detection successfully detects a marker. If no marker is detected, the tracker predicts the position. By using keypoints to detect the marker, the method accounts for in the object’s scale and rotation and can detect the marker despite motion blur. We use a moving average filter to reduce the noise in the features.

We train the classifier on manually labeled recordings of human demonstrations. Since some actions might occur less frequently than others for a given task, the dataset is balanced in advance. We compared various standard machine learning models for the experiments in this paper and report the results in Section 4.3.1.

It should be noted that the action segmentation is not the main focus of this paper but a small part of the overall developed system. The concrete classification model is interchangeable and could be replaced with more advanced methods in future work.

### 3.2 Backchaining with continous pre- and postconditions

Backchaining was proposed by Colledanchise et al. (2019) as a planning algorithm to build a BT based on pre- and post-conditions for each action. It has already been used to learn a BT from kinesthetic demonstrations based on manually defined action conditions (Gustavsson et al., 2021). The integration into a framework for interactive disambiguation based on a user’s verbal input (Iovino et al., 2022a) indicates that Backchaining is well suited for an interactive task learning setting. The use of pre- and post-conditions allows us to detect unseen states and reason about correct actions.

In contrast to other approaches that learn from human demonstrations (Colledanchise et al., 2019; Safronov et al., 2020), we use continuous pre- and post-conditions instead of only binary features. Additionally, we directly learn pre- and post-conditions for each action from human demonstrations instead of defining them manually beforehand, as in Gustavsson et al. (2021); Iovino et al. (2022a).

#### 3.2.1 Pre- and post-condition extraction from human demonstrations

In this section, we explain our approach to automatically extract pre- and post-conditions from human demonstrations in the form of RGB-D video data. Similar to the action classification (Section 3.1) we first extract object and hand positions from the demonstrations and afterwards compute pre- and post conditions from *K* pre-defined visual features f∈F based on object-object and object-hand distances. We remove feature value outliers by applying value constraints *ω*_1_(*f*), *ω*_2_(*f*) and removing all values below the 5^*th*^ and above the 95th percentile. Outliers can be caused by inaccuracies in the object and hand tracking.

From the action classifier, we obtain the high-level action sequences $a_{0}^{d},\ldots ,a_{N^{d}}^{d}$ for all human demonstrations d∈D and define O as the set of all shown actions.

For each action a∈O , we define pre- and post-conditions as value ranges between minimum values $c_{\mathrm{pre}}^{-}\left(a,f\right),c_{\mathrm{post}}^{-}\left(a,f\right)$ and maximum values $c_{\mathrm{pre}}^{+}\left(a,f\right),c_{\mathrm{post}}^{+}\left(a,f\right)$ for a feature *f* and action *a*

$$\begin{array}{cc} C_{\mathrm{pre}}\left(a\right):= & \left\{\left[c_{\mathrm{pre}}^{-}\left(a,f\right),c_{\mathrm{pre}}^{+}\left(a,f\right)\right]|f\in F_{\mathrm{pre}}\left(a\right)\right\}\\ C_{\mathrm{post}}\left(a\right):= & \left\{\left[c_{\mathrm{post}}^{-}\left(a,f\right),c_{\mathrm{pre}}^{+}\left(a,f\right)\right]|f\in F_{\mathrm{post}}\left(a\right)\right\}, \end{array}$$ (2)

where $F_{\mathrm{pre}}\left(a\right)$ and $F_{\mathrm{post}}\left(a\right)$ are feature subsets used as pre- and post-conditions for an action *a*. A condition is true if all features lie within the condition ranges. A pre-condition has to be true before action execution and is checked during action execution to allow reactivity of the corresponding BT node. Post-conditions specify which and to what range an action changes a particular feature. While we specify pre-conditions for all features $\left(F_{\mathrm{pre}}\left(a\right)=F\right)$ , the number of post-conditions can vary since most actions affect only a subset of all features $\left(F_{\mathrm{post}}\left(a\right)\subseteq F\right)$ .

In order to decide which features *f* should be included in $F_{\mathrm{post}}\left(a\right)$ for each action *a*, we calculate three metrics m_1_ (*a*, *f*), m_2_ (*a*, *f*), m_3_ (*a*, *f*) based on the corresponding set of feature value sequences ${\left\{\Psi^{a,f,\upsilon }\right\}}_{\upsilon =1,\ldots ,{ϒ}_{a}}$ of all ϒ_*a*_ action occurrences of a specific action *a* over all demonstrations. Each feature value sequence consists of values $\Psi^{a,f,\upsilon }:=\left\{\psi_{0}^{a,f,\upsilon },.,\psi_{P_{\upsilon }}^{a,f,\upsilon }\right\}$ from the start frame to the end frame of the action occurrence *υ* for each feature *f*. The intuition behind these three metrics is to use the variance in features to decide whether a feature is changed by an action inspired by Abdo et al. (2013).

First, we compute the mean difference of start and end values of all feature sequences **Ψ** ^*a*,*f*,*υ*^ over all ϒ_*a*_ action occurrences

$$m_{1}\left(a,f\right)=\frac{1}{{ϒ}_{a}}\underset{1\leq \upsilon \leq {ϒ}_{a}}{\sum }\left|\psi_{P_{\upsilon }}^{a,f,\upsilon },-,\psi_{0}^{a,f,\upsilon }\right|,$$ (3)

where $\psi_{0}^{a,f,\upsilon }$ and $\psi_{P_{\upsilon }}^{a,f,\upsilon }$ are the start and end values for feature *f* and action occurrence *a*_*υ*_.

Second, we compute the mean number of frames where the feature value change exceeds a threshold based on the minimum and maximum value of this feature over all action occurrences

$$\begin{array}{cc} m_{2}\left(a,f\right) & =\frac{1}{{ϒ}_{a}}\underset{1\leq \upsilon \leq {ϒ}_{a}}{\sum }|K\left(a,f,\upsilon \right)|\\ \;\text{where }K\left(a,f,\upsilon \right) & :=\left\{\left|i,\frac{\psi_{i+1}^{a,f,\upsilon }-\psi_{i-1}^{a,f,\upsilon }}{2}\right.\right.\\ & \;\left.>\frac{1}{\underset{1\leq \bar{\upsilon }\leq {ϒ}_{a}}{\max}\left\{\underset{j}{\max}\psi_{j}^{a,f,\bar{\upsilon }}\right\}-\underset{1\leq \bar{\upsilon }\leq {ϒ}_{a}}{\min}\left\{\underset{j}{\min}\psi_{j}^{a,f,\bar{\upsilon }}\right\}}\right\}. \end{array}$$ (4)

Third, we compute the variance of end values over all demonstrations

$$\begin{array}{cc} m_{3}\left(a,f\right) & =\mathrm{Var}\left[L\left(a,f\right)\right]\\ \;\text{where }L\left(a,f\right): & =\left\{\psi_{P_{\upsilon }}^{a,f,\upsilon }|\upsilon \in \left[1,{ϒ}_{a}\right]\right\}. \end{array}$$ (5)

Based on these three metrics, we decide whether a feature is a relevant post-condition of an action and should be included in $F_{\mathrm{post}}\left(a\right)$ according to

$$\begin{array}{cc} F_{\mathrm{post}}\left(a\right) & :=\left\{f\in F|m_{1}\left(a,f\right)>m_{1}^{-}\wedge \right.\left.m_{2}^{-}<m_{2}\left(a,f\right)<m_{2}^{+}\wedge \right.\\ & \;\;\left.m_{3}\left(a,f\right)<m_{3}^{+}\right\}, \end{array}$$ (6)

where $m_{1}^{-}$ , $m_{2}^{-}$ , $m_{2}^{+}$ and $m_{3}^{+}$ are hand-tuned thresholds. For the experiments in this paper, we set $m_{1}^{-}=0.33$ , $m_{2}^{-}=0.2$ , $m_{2}^{+}=0.8$ , and $m_{3}^{+}=0.2$ .

For each action and feature in $F_{\mathrm{pre}}\left(a\right)$ and $F_{\mathrm{post}}\left(a\right)$ , we now want to define minimum and maximum values $c_{\mathrm{pre}}^{-}\left(a,f\right),c_{\mathrm{pre}}^{+}\left(a,f\right),c_{\mathrm{post}}^{-}\left(a,f\right),c_{\mathrm{post}}^{+}\left(a,f\right)$ for the condition ranges in $C_{\mathrm{pre}}\left(a\right)$ , and $C_{\mathrm{post}}\left(a\right)$ .

For a post-condition for a feature *f* and action *a*, we consider the last *δ* frames over all action occurrences and the remaining frames for the corresponding pre-condition. In our experiments, we use *δ* = 3. We compute minimum and maximum values for the condition ranges $C_{\mathrm{pre}}\left(a\right),C_{\mathrm{post}}\left(a\right)$ over these feature values

$$\begin{array}{cc} c_{\mathrm{pre}}^{-}\left(a,f\right)= & \min\left(\left\{\psi_{0:P_{\upsilon }-\delta -1}^{a,f,\upsilon }|\upsilon \in \left[1,{ϒ}_{a}\right]\right\}\right)\text{ for }f\in F_{\mathrm{pre}}\left(a\right)\\ c_{\mathrm{pre}}^{+}\left(a,f\right)= & \max\left(\left\{\psi_{0:P_{\upsilon }-\delta -1}^{a,f,\upsilon }|\upsilon \in \left[1,{ϒ}_{a}\right]\right\}\right)\text{ for }f\in F_{\mathrm{pre}}\left(a\right)\\ c_{\mathrm{post}}^{-}\left(a,f\right)= & \min\left(\left\{\psi_{P_{\upsilon }-\delta :P_{\upsilon }}^{a,f,\upsilon }|\upsilon \in \left[1,{ϒ}_{a}\right]\right\}\right)\text{ for }f\in F_{\mathrm{post}}\left(a\right)\\ c_{\mathrm{post}}^{+}\left(a,f\right)= & \max\left(\left\{\psi_{P_{\upsilon }-\delta :P_{\upsilon }}^{a,f,\upsilon }|\upsilon \in \left[1,{ϒ}_{a}\right]\right\}\right)\text{ for }f\in F_{\mathrm{post}}\left(a\right). \end{array}$$ (7)

We post-process these ranges $C_{\mathrm{pre}}\left(a\right),C_{\mathrm{post}}\left(a\right)$ , so that the pre- and post-conditions of an action for a given feature do not overlap and that all initial ranges are not smaller than a predefined threshold *τ*(*f*) (in our experiments, we use *τ*(*f*) = 1.5 *cm* as a threshold for all features except the finger distance).

At last, the conditions have to be adapted so that the pre- and post-conditions of adjacent actions fit together. Algorithm 1 summarizes the details of the entire condition computation.

Algorithm 1 Condition Computation.

**Require:** O : action shown in human demonstrations, F : features, D : demonstrations

$\tau \left(f\right),\zeta ,\omega_{1}\left(f\right),\omega_{2}\left(f\right)\text{ for }f\in F:\text{parameters}$

 **for** a∈O **do**
  **for** f∈F **do**
   Remove outliers
   Calculate metrics m_1_ (*a*, *f*), m_2_ (*a*, *f*), m_3_ (*a*, *f*) (Equation 3, 4, 5)
   Determine relevant post-conditions $F_{\mathrm{post}}\left(a\right)$ for each action *a* according to Equation 6
  **end for**
  
$C_{\mathrm{pre}}\left(a\right):=\left\{\left[c_{\mathrm{pre}}^{-}\left(a,f\right),c_{\mathrm{pre}}^{+}\left(a,f\right)\right]|f\in F_{\mathrm{pre}}\left(a\right)\right\}$ ⊳ Define condition ranges (Equation 7)
  
$C_{\mathrm{post}}\left(a\right):=\left\{\left[c_{\mathrm{pre}}^{-}\left(a,f\right),c_{\mathrm{pre}}^{+}\left(a,f\right)\right]|f\in F_{\mathrm{post}}\left(a\right)\right\}$

  Adapt conditions so that pre- and post-conditions do not overlap
  Widen small condition ranges below a value difference *τ*(*f*)
 **end for**
 **for** *d* ∈ *D* **do**
  **for** *n*_*d*_ ∈ [0, *N* ^*d*^] **do** ⊳ Adapt conditions of adjacent actions
   
$a\leftarrow a_{n_{d}}^{d}$

   
$\hat{a}\leftarrow a_{n_{d}+1}^{d}$

   **for** $f\in F_{\mathrm{post}}\left(\hat{a}\right)$ **do**
    **if** $notf\in F_{\mathrm{post}}\left(a\right)$ **then**
     
$c_{\mathrm{pre}}^{-}\left(\hat{a},f\right)=\min\left(c_{\mathrm{pre}}^{-}\left(\hat{a},f\right),c_{\mathrm{pre}}^{-}\left(a,f\right)\right)$

     
$c_{\mathrm{pre}}^{+}\left(\hat{a},f\right)=\max\left(c_{\mathrm{pre}}^{+}\left(\hat{a},f\right),c_{\mathrm{pre}}^{+}\left(a,f\right)\right)$

    **else**
     
$c_{\mathrm{pre}}^{-}\left(\hat{a},f\right)=\min\left(c_{\mathrm{pre}}^{-}\left(\hat{a},f\right),c_{\mathrm{post}}^{-}\left(a,f\right)\right)$

     
$c_{\mathrm{pre}}^{+}\left(\hat{a},f\right)=\max\left(c_{\mathrm{pre}}^{+}\left(\hat{a},f\right),c_{\mathrm{post}}^{+}\left(a,f\right)\right)$

    **end if**
   **end for**
  **end for**
 **end for**
 **for** *d* ∈ *D* **do**
  
$S\left(d\right):=\left\{\left[c_{\mathrm{pre}}^{-}\left(a_{0}^{d},f\right),c_{\mathrm{pre}}^{+}\left(a_{0}^{d},f\right)\right]|f\in F_{\mathrm{pre}}\right\}$ ⊳ Define start conditions
 **end for**

$G:=\left\{\left[c_{\mathrm{post}}^{-}\left(a_{n_{d}}^{0},f\right),c_{\mathrm{post}}^{+}\left(a_{n_{d}}^{0},f\right)\right]|f\in F_{\mathrm{post}}\right\}$ ⊳ Define goal conditions

#### 3.2.2 Backchaining

The extracted pre- and post-conditions for each action, together with the action sequences of all demonstrations, are used to construct an initial BT using the Backchaining algorithm (Colledanchise et al., 2019). First, we define a goal condition G as the post-condition ranges of the last shown action of the demonstrations, assuming that the goal state is identical for all demonstrations for a particular task. This goal condition is placed at the root sequence of the tree. The tree is then iteratively searched for a failing condition using self-simulation as proposed by Safronov et al. (2020), starting iteratively from all start condition ranges S(d) of all demonstrations. Here, the start condition S(d) is a set of pre-condition ranges of the first action of each demonstration *d*. In each step, a subtree whose post-condition satisfies the failed pre-condition replaces this condition until the goal condition is reached. However, the generated order might result in logical conflicts. There might be conflicting conditions in the same path of the tree resulting in, e.g., a gripper that is supposed to be closed and opened simultaneously. Since this can never be fulfilled, such conflicts need to be resolved. We use a conflict-resolving strategy adapted from Safronov et al. (2020). The conflicting subtree is moved leftwards and upwards until the conflict is resolved. Lastly, the resulting BT can be pruned by removing unnecessary conditions. An overview of the Backchaining algorithm is given in Algorithm 2.

Algorithm 2 Backchaining Algorithm (adapted from Safronov et al. (2020))

**while** G≠True **do**
 **for** d∈D **do**
  Search for failing condition by self-simulation starting with S(d)

  Search action that satisfies the failed condition
  Replace failed condition with subtree
  Search and fix potential conflicts
 **end for**
**end while**
Prune unnecessary nodes

We learn reactive behavior trees that can solve the given task despite external influences. Therefore, the learned BT is ticked regularly with a given frequency. The generic BT actions must return *RUNNING* while a lengthy action is performed. If an action returns *RUNNING*, the tick is propagated upwards to the root node, and the tree is ticked again. In this way, the feature state can be continuously monitored and the executed action can be changed if the pre-conditions are no longer fulfilled. In this way, safety checks, e.g., that no human is too close to the robot can be easily integrated.

### 3.3 Interactive handling of failure cases

We propose a method of learning a BT from only a few human demonstrations. As a result of this, the user effort is kept low, and after only a short training phase, the robot is already able to execute the initial BT. However, few demonstrations might not cover all possible scenarios or failure cases, and handling such incomplete demonstrations is a challenge (Gustavsson et al., 2021).

In a study with 22 participants on how non-expert users demonstrate a robotic task (Section 4.2), we identified three main problems when executing BTs learned from such imperfect human demonstrations. In the following, we shortly describe these potential failure scenarios and explain how we propose to resolve them automatically in our interactive approach. An overview of all failure cases and how they are resolved based on the user input is shown in Figure 8.

#### 3.3.1 Resolving a pre-condition failure

We detect failure cases as either failing pre-conditions before and during an action or failing post-conditions after the execution of an action (yellow in Figure 8). A condition fails if the corresponding feature does not lie within the defined value range.

There are two reasons why a pre-condition could fail: Either the robot is trying to execute the correct action, but the pre-conditions of this action do not include the current situation, or a suboptimal post-condition lead to a wrongly ticked action in the BT. If an action is, for example, already successfully executed but the post-condition is suboptimal and therefore not fulfilled, the robot could still try to execute this action. In order to decide how to resolve the situation, the system explains what actions it is trying to perform next to check if the correct subtree in the BT is ticked. The user is asked if the robot is indeed pursuing the correct actions. If the user does not confirm, the robot asks which action must be performed instead. In this case, a post-condition must have been learned incorrectly (blue in Figure 8). Given the current feature values ${\hat{\psi }}_{\mathrm{cur}}^{f}$ and the correct action, it is possible to backtrack the BT and identify the suboptimal post-condition. This post-condition is changed in a way that the tick would have ended up in the suggested action according to the current feature values. Here, each feature range in the post-condition is compared with the current feature values. If the value exceeds the maximum value of the post-condition range $c_{\mathrm{post}}^{-}\left(a,f\right)$ , it is set to the current feature value increased by the parameter *ϵ*: $c_{\mathrm{post}}^{+}\left(a,f\right)=\left(1+\epsilon \right)\cdot {\hat{\psi }}_{\mathrm{cur}}^{f}$ . The same applies for the minimum value of the condition $c_{\mathrm{post}}^{-}\left(a,f\right)$ if the current feature value is lower: $c_{\mathrm{post}}^{-}\left(a,f\right)=\left(1-\epsilon \right)\cdot {\hat{\psi }}_{\mathrm{cur}}^{f}$ . In our experimental evaluation *ϵ* is set to 0.1. The pre- and post-condition adaptation is summarized in Algorithm 3.

Algorithm 3 Pre-condition and Post-condition Adaptation

**Require:** *ϵ*: parameter increasing the amount of change
 **if** $c_{\mathrm{pre∕post}}^{-}\left(a,f\right)>{\hat{\psi }}_{\mathrm{cur}}^{f}$ **then**
  
$c_{\mathrm{pre∕post}}^{-}\left(a,f\right)=\left(1-\epsilon \right)\cdot {\hat{\psi }}_{\mathrm{cur}}^{f}$

 **else if** $c_{\mathrm{pre∕post}}^{-}\left(a,f\right)<{\hat{\psi }}_{\mathrm{cur}}^{f}$ **then**
  
$c_{\mathrm{pre∕post}}^{+}\left(a,f\right)=\left(1+\epsilon \right)\cdot {\hat{\psi }}_{\mathrm{cur}}^{f}$

 **end** **if**

If the user confirms the robot’s next planned actions, an existing pre-condition must be extended to include the current state (magenta in Figure 8). This scenario can occur if the human demonstrations of the currently correct action did not include the current feature state. If a user demonstrates the *Move-to-Trash* action for only one position of the trash, the pre-conditions of this action would, for example, fail for a different trash positioning for the feature *dis_trash_trashcan*. Since the robot knows which condition is failing, it can suggest the most helpful action based on the pre- and post-conditions. The user either confirms this action or selects a different action, and the range of the failed pre-condition of this action is adapted according to Algorithm 3. Similar to the adaptation of post-conditions, the minimum and maximum condition values $c_{\mathrm{pre}}^{-}\left(a,f\right),c_{\mathrm{pre}}^{+}\left(a,f\right)$ are increased or decreased if the current feature value ${\hat{\psi }}_{\mathrm{cur}}^{f}$ is lower or higher in comparison. If it is lower, $c_{\mathrm{pre}}^{-}\left(a,f\right)$ is set to $\left(1-\epsilon \right)\cdot {\hat{\psi }}_{\mathrm{cur}}^{f}$ and if it is higher $c_{\mathrm{pre}}^{+}\left(a,f\right)$ is set to $\left(1+\epsilon \right)\cdot {\hat{\psi }}_{\mathrm{cur}}^{f}$ where *ϵ* is a parameter used to increase the change of the adapted range limit. Inaccuracies in the features can otherwise lead to repeated failures during execution because a feature keeps exceeding the range limits and is only marginally changed in each step.

#### 3.3.2 Resolving a post-condition failure

After an action succeeds and returns *RUNNING*, we check if the resulting feature state satisfies the learned post-conditions of this action. Due to differences between the human demonstrations and the robot’s action execution, the action-outcome might not match the learned post-conditions. In this case, the user is asked whether the last action has been successfully executed and adjusts the post-condition based on the current state according to Algorithm 3 (blue in Figure 8). If the action *Release-Trash* is, for example, successfully executed but the resulting distance between the robot grippers does not match the finger distance shown in the human demonstrations, the post-condition regarding this feature would fail.

#### 3.3.3 Resolving unseen situations with additional demonstrations

In case a pre-condition fails, it may happen that the robot can not resolve the situation with any of the demonstrated actions so far (green in Figure 8). This may happen if a user demonstrates a pick-and-place task where the object is well-placed for grasping but during robot execution another object is placed on top of this object and first has to be put aside to fulfill the task. The learned BT would then fail since this situation and the required actions were not shown in the human demonstrations. In this case, the user can show additional demonstrations of the required action or sequence of actions until the previously failed pre-condition in the initially learned BT is satisfied. Those demonstrations are used to compute conditions for the new actions as described in Section 3.2.1. The failed pre-condition is then replaced iteratively by the newly demonstrated actions using the Backchaining approach (Section 3.2.2) until the failed pre-condition is satisfied.

After an adaptation of the learned BT and corresponding conditions as described in the previous sections, it is necessary to make sure that all pre- and post-conditions of adjacent actions still fit together and, if necessary, adapt them accordingly (Algorithm 1).

It should be noted that rebuilding the entire tree is not necessary to resolve the described failure cases. Instead, the failing condition is adapted or replaced by a subtree. As a result, the BT grows, and conditions are refined as the robot deals with new situations, but the initial BT structure remains unchanged. Here, our approach avoids repeating a similar demonstration multiple times since only local changes are required, and the previously learned BT is exploited.

## 4 Experimental evaluation on a robotic trash disposal task

We evaluate our method on a robotic trash disposal task with a Franka Erika Panda robot arm. In a pilot study with 22 participants, we analyze what kind of task demonstrations non-expert users provide and what failure cases occur when executing a BT learned from these potentially imperfect and incomplete demonstrations. In a second study, we evaluate our resulting overall system, including interactive resolving of failure cases at execution time of the BT with 20 human participants. We use the demonstration data from the pilot study to train an action classifier that predicts high-level action sequences from RGB-D video recordings of human demonstrations. In the following, we first describe the experiment setup in detail in Section 4.1. Afterwards, in Section 4.2, we analyze the human demonstrations from the pilot study. Lastly, we evaluate the overall interactive approach and analyze user satisfaction regarding the overall system in Section 4.3.

### 4.1 Trash disposal task setup

**FIGURE 2:**
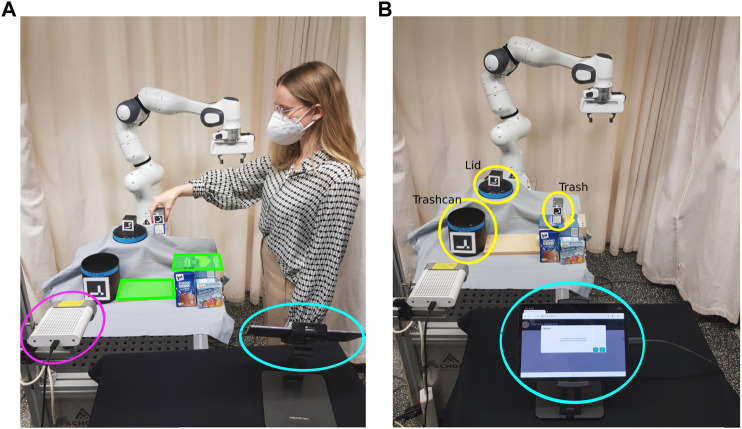
**(A)** A participant is demonstrating the task. The demonstration recording can be started using the web-interface on the tablet (blue). The demonstration is recorded by the Kinect Azure camera (magenta). Before the start of a demonstration, the participant is asked to place the trash somewhere in the area marked in green. **(B)** The robot is executing the task while the user provides input via the tablet (blue). The task-relevant objects (lid, trashcan, trash) are highlighted in yellow.

Figure 2 shows the experiment setup for the trash disposal task. The robot is supposed to learn how to pick up trash (empty tetra-pack) placed in the area marked in green Figure 2A and dump it in a trashcan. If the lid is placed on top of the trashcan, the robot should learn how first to put the lid aside. The objects trash, trashcan, and lid are highlighted in yellow in Figure 2B. An Azure Kinect RGB-D camera (magenta in Figure 2A) is used to record all human demonstrations and obtain RGB and depth information about the scene.In order to learn a task representation in the form of a BT, we map the human’s high-level actions to the robot’s pre-defined high-level actions. The set of actions consists of *Move-to-Trash*, *Grasp-Trash*, *Move-to-Trashcan*, *Release-Trash*, *Move-to-Lid*, *Grasp-Lid*, *Move-to-Drop-Off*, *Release-Lid*. All actions are implemented as custom reactive action nodes in the BehaviorTree.CPP framework (Faconti, 2018). Action nodes frequently return RUNNING before they finish and either return SUCCESS or FAILURE in order to be able to react to external changes. The user can communicate with the system via a web-interface on a tablet (highlighted in blue in Figure 2). This web-interface is used for the recording of the demonstrations (Figure 2A) and for interactive handling of failure cases during robot execution of the initially learned BT (Figure 2B). The web-interface dialogue is shown in Figure 8.

### 4.2 Pilot study to identify imperfections in human demonstrations

In related approaches that learn BTs from human demonstrations, there is a lack of experimental analysis on how non-experts actually demonstrate tasks and what could be potential pitfalls when learning BTs from such demonstrations. Experiments are either conducted with a user familiar with the system (Helenon et al., 2021; Iovino et al., 2022b) or only consider natural language instructions (Suddrey et al., 2022) or kinesthetic teaching (Gustavsson et al., 2021) but no human demonstrations of complete task sequences. However, we consider it crucial to use insights about how people demonstrate tasks to handle imperfect demonstrations. In order to investigate what kind of task demonstrations non-expert users provide to our robot and to analyze possible failure cases of BTs learned from such demonstrations, we asked 22 participants (9 male, 13 female) to demonstrate the trash disposal task, as described in Section 4.1. In the beginning, we gave the participants written instructions explaining the general experiment setup and procedure. We asked them to demonstrate the task with slow movements and only use their right hand. Each participant was instructed to demonstrate the task three times and *vary* the demonstrations in between these three trials. We did not explicitly state how they should vary the demonstrations.

Not only for learning BTs from demonstrations but also for most other LfD approaches, variations are essential to learn meaningful task representations that generalize well to different situations (Abdo et al., 2013; Knaust and Koert, 2021). With our experiment, we contribute an analysis that provides insights on how non-expert users vary their demonstrations and discuss potential failure cases that could occur when learning a BT from these demonstrations using the approach described in Section 3.2.

Before each demonstration, we asked the participants to place the trash somewhere in the area marked in green, shown in Figure 2A, and then start the demonstration using the web-interface (Figure 8A). The demonstrations were recorded with a framerate of 30fps by an Azure Kinect RGB-D highlighted in magenta in Figure 2.

#### 4.2.1 Analysis of variations in human demonstrations

We analyze variations in the human demonstrations based on recorded object positions and a questionnaire that participants answered after providing the demonstrations.

**FIGURE 3:**
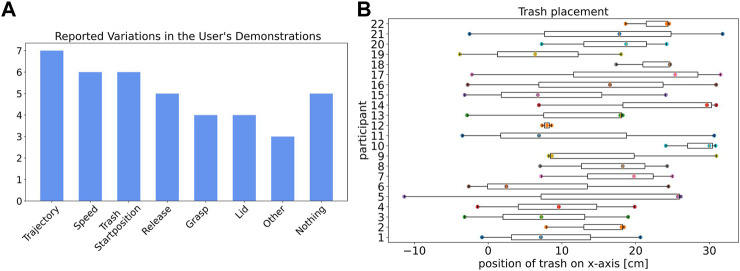
**(A)** Reported ways the participants tried to vary their task demonstrations. Some participants reported multiple types of variation. **(B)** Trash placement along the *x*-axis before all three demonstrations over all participants.

The reported variations based on the questionnaire are shown in Figure 3A. Five of all 20 participants stated that they did not intentionally vary their demonstrations since they either forgot this request or did not know what to vary. The other participants reported different ways of varying their demonstrations, which can be grouped into seven categories. Seven participants demonstrated different trajectories when they reached out to the trash and then moved the trash to the trashcan. Six participants reported variations in speed and trash placement. Nine participants changed the way they grasped and released the trash over their demonstrations. Only four participants stated that they varied their demonstrations by incorporating the lid of the trashcan in some of their demonstrations, either placing the lid on the trashcan before a demonstration or placing it on top of the trashcan after they put the trash in the trashcan. Some variations were only shown by one participant, such as varying the height of the trash when releasing it or deliberately hitting the trashcan with the trash before correctly releasing it in the trashcan to show the robot the position of the trashcan.

The positioning of the trash for all participants along the *x*-axis is shown in Figure 3B. Participants 11, 14, 15, 16, 19, and 21 reported that they intentionally varied the trash positioning within their three demonstrations. Particular notable, there are also subjects that did not report a variation in the trash positioning and still varied the positions (e.g., participant 5). Some subjects only marginally changed the trash placement with position differences below 10 cm within the demonstrations (e.g., participants 10, 12, 22).

We identified three prominent failure cases that can occur when executing a BT learned directly from recorded human demonstrations, as described in Section 3.2. First, showing only minor variations in the demonstration can lead to failures or unknown situations when the robot executes the initial BT learned from a few demonstrations. Suppose the user, for instance, only marginally changes the trash placement for all demonstrations. In that case, the condition ranges will only cover this specific case and lead to a pre-condition failure if the trash is placed slightly more to the left or right. The same applies for variations in the movements and usage of the lid.

To be able to handle such pre-condition failure cases during execution time and still be able to learn from only a few human demonstrations, we propose interactively refining failing pre-conditions as described in Section 3.3.1.

A second failure case can occur caused by differences in the demonstrated actions and corresponding robotic actions. If the demonstrations of the action *Move-to-Trashcan*, for example, always end around a certain position above the trashcan and this end position differs for the robotic action, the learned post-condition of this action fails during execution. In this case, the post-condition of this action has to be refined as described in Section 3.3.2.

Since only four out of all 20 participants included the lid of the trashcan in their demonstrations, the learned BT would fail for all other participants in situations were the lid is placed on top of the trashcan. In this case, the required action sequence to first put the lid aside is not shown in the demonstrations. In such cases where required actions were not shown in the demonstrations used to learn the initial BT, we propose extending the BT according to additional human demonstrations of those actions (Section 3.3.3).

In pilot tests, we observed that some users only performed movements along one axis resulting in right-angled movement, presumably to imitate a robot and support learning of the task by the robot. We suspect that users intentionally adapt their demonstrations if they are aware that those demonstrations are used to teach a robot a task. To gain deeper insights into this phenomenon, after the experiment, we specifically asked the participants in a questionnaire whether they demonstrated the task how they would usually perform it or if they demonstrated the task as they expected the robot to perform the task. Out of all 22 participants, 12 users stated that they performed the task as they would usually perform it. On the other hand, ten subjects reported adapting their demonstrations to the expected robot behavior.

### 4.3 Evaluation of interactive BT learning on robotic trash disposal task

We conducted robotic experiments with 20 participants (8 male, 12 female) to evaluate the overall system, including the pipeline to learn an initial BT directly from a few human video demonstrations and interactive refinement of this initially learned BT during robot execution. Here, we use a classifier trained on the demonstration data collected in the pilot study described in Section 3.1 for action segmentation.

Of all 20 participants, 15 were between 18 and 25 years old and five between 26 and 35 years old. The subjects mainly reported a low level of prior experience with robots. In particular, eleven persons never had direct contact with robots before, five persons reported less than ten encounters with robots, and only five persons had contact with robots more than ten times.

The experiment can be divided into two parts. First, the participants showed three task demonstrations, and an initial BT was built as described in Section 3.2. Afterwards, the robot executed the BT, and the participants should interactively resolve eventually occurring failure cases. In the following, we first present the results of the action segmentation trained on the dataset collected in the pilot study (Section 4.3.1). Then, we analyze the results of the action segmentation, pre- and post-condition computation, and BT building in Section 4.3.2. Section 4.3.3 evaluates all failure cases during execution and how those failures are resolved. The user satisfaction regarding the interaction with the overall system according to the User Experience Questionnaire (UEQ) is analyzed in Section 4.3.4. The reactivity of the learned BTs is showcased in Section 4.3.5, and an example of how additional demonstrations can be used to extend an initial BT for the trash disposal task is described in Section 4.3.6.

#### 4.3.1 Training of classifier for action segmentation

On the recorded dataset, we compare different supervised standard machine learning models for action segmentation as extracting the demonstrated action sequence is a necessary part of the overall developed pipeline. In addition, we report challenges encountered when segmenting pick-and-place actions from human demonstrations.

As described in Section 4.2, in the pilot study the subjects first demonstrated the task three times without further instructions on how to perform the task precisely. After they completed these three trials, we additionally collected demonstrations with more detailed instructions in order to have a well-structured dataset to train an action classifier for the trash disposal task. Here, we first asked them to demonstrate the task three times from pre-defined varying starting positions of the trash without moving the trash lid. Afterwards, we specifically asked them to first place the lid on the trashcan before demonstrating the task again three times.

As features for the classifier, we used the distances between hand to trash, hand to trashcan, trash to trashcan, thumb to index-finger, hand to lid, trash to lid, trashcan to lid, and the velocity of the trash. All distances and velocities are computed based on the extracted object positions and hand features, as described in Section 3.1.

A rolling window of five frames before and five frames after each frame was used. We manually labeled the videos of human demonstrations to obtain the ground truth labels. The action classification results for all models are shown in Table 1. We trained the models in a k-fold cross-validation fashion on the balanced dataset of 21 participants and used the remaining participant for validation. We report the mean of the model scores over all 22 folds. The best results are achieved using a Support Vector Machine with a polynomial kernel of degree 3, as highlighted in Table 1. It achieves a weighted accuracy score of 0.849, F1-score of 0.856, precision of 0.843, and recall of 0.897. The precision using a Random Forest model is slightly higher with 0.856. However, the Random Forest model only achieves an accuracy of 0.725 on the validation dataset.

**TABLE 1:** Action segmentation results on the prestudy dataset for different models.

Model	Acc. Train	Acc. Test	F1 train	F1 test	Precision	Recall
Logistic Regression	0.824	0.800	0.802	0.795	0.771	0.871
Perceptron	0.777	0.754	0.775	0.752	0.735	0.869
Linear SVC	0.839	0.812	0.811	0.803	0.779	0.877
SVC Polynomial Kernel 3	0.913	**0.849**	0.894	**0.856**	0.843	**0.897**
KNN	0.985	0.751	0.971	0.841	0.833	0.864
Decision Tree	1.0	0.666	1.0	0.801	0.802	0.821
Random Forest	1.0	0.725	1.0	0.853	**0.856**	0.867
MLP	0.951	0.797	0.937	0.853	0.845	0.883

The bold values highlight the highest value achieved for each evaluation metric.

Throughout the pilot study, we observed two general problems that can occur when learning a robotic pick-and-place task from human demonstrations. First, humans tend to only marginally open and close their fingers when demonstrating grasp or release action. This makes it difficult to reliably detect those actions and learn meaningful conditions based on the distance between thumb and index-finger. Second, without a fixed starting and end position for the user’s hand, the user might unintentionally skip actions that are necessary for the robot to perform the task. Some participants already placed their hand above the trash at the beginning of their demonstration so that the action *Move-to-Trash* is not shown. However, the robot must first execute the action *Move-to-Trash* to reach this position above the trash from its start position. To avoid these problems, we adapted the written instructions about demonstrations before the second study. Specifically, we ask the participants to keep their hand wide open unless they are grasping the trash and include a fixed start and end position of the user’s hand for the demonstrations. However, it should be noted that these modifications of the instructions may constrain the users in how they demonstrate.

Since those changes in the instructions may result in slightly different demonstrations, we extended the training dataset with additional demonstrations of the task with a fixed start and end position of the hand and a wider open hand. In total, we added 20 demonstrations without usage of the lid and 22 demonstrations of the task with the lid placed on the trashcan in the beginning.

The action segmentation results trained on this extended dataset are reported in Table 2. Again, the Support Vector Machine with a polynomial kernel of degree 3 achieves the best results with a weighted accuracy score of 0.853, F1-score of 0.863, precision of 0.850, and recall of 0.901. Again, the precision of the trained Random Forest model is slightly higher with 0.861, and a Multilayer Perceptron (MLP) achieves a similar F1-score. Since, overall, the Support Vector Machine outperforms the other classifiers, we used this model trained on the extended dataset for our robotic experiments described below.

**TABLE 2:** Action segmentation results on the extended dataset for different models.

Model	Acc. Train	Acc. Test	F1 train	F1 test	Precision	Recall
Logistic Regression	0.837	0.813	0.820	0.808	0.785	0.882
Perceptron	0.781	0.752	0.792	0.772	0.752	0.870
Linear SVC	0.849	0.824	0.828	0.818	0.796	0.885
SVC Polynomial Kernel 3	0.916	**0.853**	0.900	**0.863**	0.850	**0.901**
KNN	0.982	0.766	0.967	0.843	0.835	0.867
Decision Tree	1.0	0.683	1.0	0.807	0.808	0.821
Random Forest	1.0	0.759	1.0	0.860	**0.861**	0.875
MLP	0.952	0.813	0.941	**0.863**	0.856	0.887

The bold values highlight the highest value achieved for each evaluation metric.

#### 4.3.2 Experimental evaluation of BT building from human demonstrations

**FIGURE 4:**
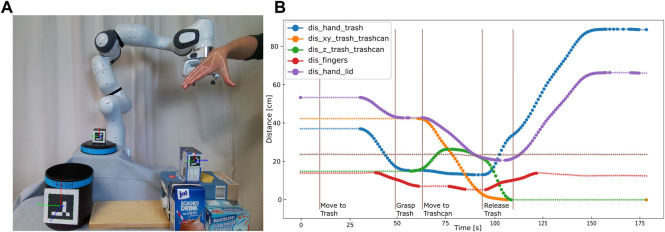
**(A)** Detected hand and object positions for the objects trash, trashcan, and lid. The participants are asked to start and end the demonstration with the hand placed close to the robot gripper. **(B)** Extracted features for one exemplary demonstration. The segmented actions using the trained classifier are shown with vertical lines.

At the beginning of the experiment, participants were given written instructions explaining the task and how to start the recording of the demonstrations using the web-interface shown in Figure 8A. Compared to the first study, they were asked to keep their hand wide open unless they grasp the trash and start and end their demonstrations with the hand placed close to the robot gripper, as shown in Figure 4A.

After reading the instructions, the participants recorded three demonstrations, from which a BT was built according to the pipeline described in Section 3. For the given task, we used the condition features described in Table 3.

**TABLE 3:** Description of all condition features used for the robotic trash disposal task.

Feature	Description
dis_xy_trashcan_lid	Distance in xy direction between trashcan and lid
dis_xy_trash_trashcan	Distance in xy direction between trash and trashcan
dis_z_trash_trashcan	Distance in height between trash and trashcan
dis_hand_trash	Distance between wrist and trash
dis_fingers	Distance between index-finger and thumb
dis_hand_lid	Distance between wrist and lid

**FIGURE 5:**
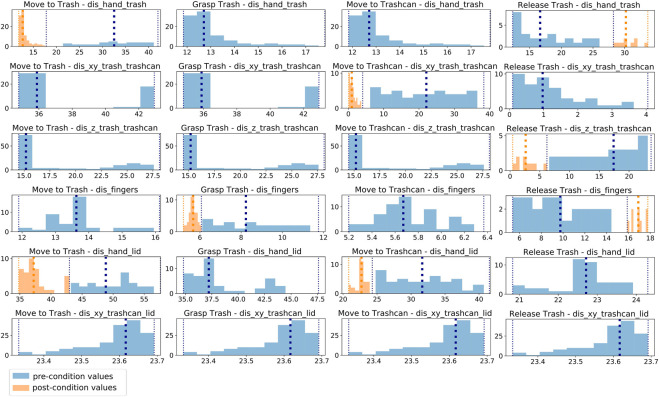
All feature values corresponding to pre-conditions (blue) and post-conditions (orange) for one participant over all actions and features. If an action changes a features, this feature is used as a post-condition for this action. The pre-conditions of all subsequent actions are adapted according to this post-condition.

Figure 4 illustrates these extracted features over a complete task demonstration for one participant. Here, the distance between the lid and the trashcan is not visualized since the lid was not used in the demonstrations, and the value is, therefore, constant. We used a moving average filter over five frames to reduce the noise in the features. One can see that during the *Move-to-Trash* action, the distance between hand and trash and hand and lid decreases. The distance between the thumb and index-finger changes for the action *Grasp-Trash*, whereas the features *dis_hand_trashcan* and *dis_hand_lid* change during the action *Move-to-Trashcan*. For the action *Release-Trash*, the distance between the thumb and index-finger changes, and also the distance between trash and trashcan and hand and trash since the trash falls down. The learned post-conditions of each action reflect the features changed by each action. The learned conditions for one participant over all actions and features are visualized in Figure 5. The feature values corresponding to a pre-condition are shown in blue and all post-conditions are shown in orange.

**FIGURE 6:**
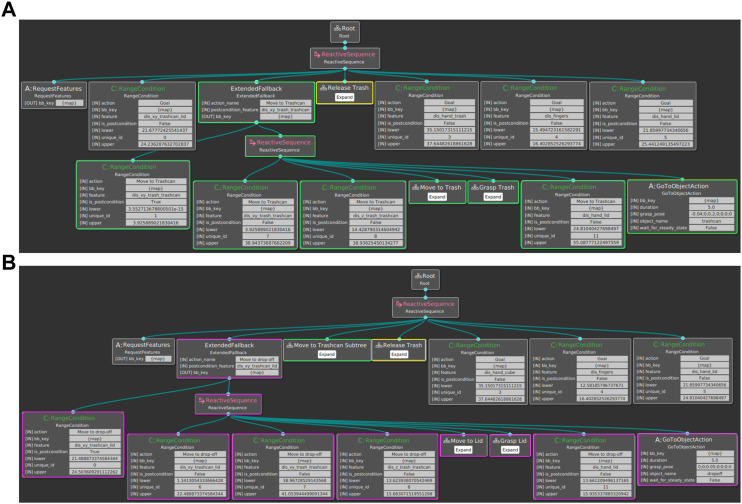
**(A)** Example of a BT built using the three demonstrations of one participant. For better readability, the subtrees of the *Move-to-Trash*, *Grasp-Trash*, and *Release-Trash* action are collapsed. Each action is included in form of a Fallback node with the post-conditions on the left and the pre-conditions and corresponding action node on the right. **(B)** Expanded BT using additional human demonstrations of how to put aside the lid before moving towards the trash. The corresponding subtrees of the initially learned BT are highlighted in green and yellow. The added subtree based on the additional demonstrations is marked in magenta.

A BT learned from the given human demonstrations is shown in Figure 6A. For better readability, the subtrees for the actions *Move-to-Trash*, *Grasp-Trash*, and *Release-Trash* are collapsed. The *RangeCondition* nodes represent the learned conditions with the respective feature and upper and lower values. During execution, the root is ticked with a frequency of 0.1 Hz. In the beginning, all features are requested in the *RequestFeatures* node. Since the distance between the trashcan and lid stayed the same for all demonstrations, the goal condition of this feature is already fulfilled in the beginning. Next, we check if the pre-condition of the action *Move-to-Trashcan* regarding the distance between trash and trashcan is already fulfilled. If the trash is still far from the trashcan, the fallback subtree is ticked. In this subtree, the pre-conditions of the action *Move-to-Trash* are first checked, and if possible, *Move-to-Trash* is executed. Then, the action *Grasp-Trash* is performed if the pre-conditions allow it. The same applies for the action *Move-to-Trashcan* and *Release-Trash*. Finally, we check if all goal conditions are fulfilled, and if they are, the execution of the BT is terminated.

**FIGURE 7:**
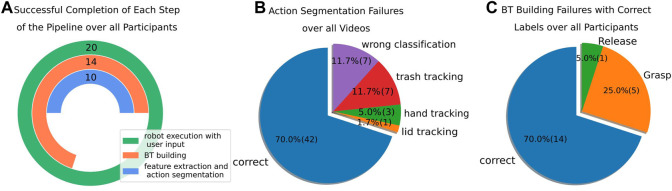
**(A)** Number of successful completions of each step of the pipeline including feature extraction and action segmentation, condition computation and BT building, and robotic task execution with user input assuming successful completion of the previous step. The number of successful completions over all 20 participants is reported. **(B)** Action segmentation failures and corresponding reasons for all 60 shown demonstrations. **(C)** Failures during condition computation and BT building assuming correct action labels. The actions causing problems are reported.

In Figure 7A, we analyze the number of successful completions of each step of the pipeline, including feature extraction and action segmentation, condition computation and BT building, as well as robot execution of the learned BT and interactive resolvent of failure cases. In case a BT was successfully built from the human demonstrations, all failure cases during execution could be solved based on the user input, and the robot could successfully finish the task for those learned BTs. However, the feature extraction and action segmentation results failed for 10 participants, which hindered the condition computation and BT building in those cases. Using correct labels from manual annotations, the condition computation and BT building were successful for 14 participants. In order to be able to test the interactive refinement through user communication for all participants, we used a fallback BT in the experiments in case no BT could be built. The robot execution of the learned BT and interactive resolvent of failure cases was successful for all 20 participants. In the following, the failures during the different steps of the pipeline and corresponding reasons are analyzed in more detail.

For the 10 participants, the action classifier did not extract the correct action sequence in at least one of the three demonstrations. The confusion matrix for the action segmentation over all demonstrations is shown in Figure 9. Adjacent actions such as *Move-to-Trash* and *Grasp Trash* are often confused since it is hard to tell in which frame exactly an action starts and another action ends. Reasons for problematic action segmentation failures are broken down in Figure 7B. The hand or object detection failed for eleven demonstrations, resulting in partially wrong features and incorrectly predicted actions. In particular, one participant wore a long-sleeved t-shirt which hindered Media Pipe’s hand tracking at the beginning of all three demonstrations. In seven of all 60 demonstrations, the trash could not be detected correctly for all frames, and in one demonstration the lid detection failed. Those errors in object detection often occurred at the end of the demonstration, which is also reflected in a high confusion of the actions *Nothing* as *Move-to-Trash*. Especially the detection of the trash after the action *Release-Trash* was problematic since the ArUco marker was not visible inside the trashcan and in this case often detected at an incorrect position. For seven demonstrations, the pre-trained action classifier assigned incorrect labels despite an accurate feature extraction. For all cases where at least one demonstration was incorrectly labeled, the conditions could not be learned correctly and the BT could not be built. However, for all cases where the correct action sequence was predicted, meaningful conditions could be extracted and an executable BT was successfully built.

In order to be able to test the interaction during robot execution for all participants, a fallback BT was used in case the BT could not be built. Figure 7C illustrates how often a BT could be built successfully in case we use manually annotated action labels. In this case, meaningful conditions could be extracted, and a BT was built for 14 of all 20 participants. For five participants, the finger distance is not recognized as a relevant post-condition for the action *Grasp-Trash* resulting in a BT only consisting of the actions *Move-to-Trash*, *Move-to-Target*, and *Release-Trash*. For one participant, the same problem occurs for the *Release-Trash* action.

#### 4.3.3 Experimental evaluation of interactive failure case handling

The goal of the second part of the study was to evaluate if the robot can successfully execute the learned BT and if failure cases during execution can be resolved by refining this initial BT with the help of the user.

For the robot execution, we replace the wrist position of the user with the position of the robot’s end-effector minus a small offset. The index-finger and thumb position are replaced with both gripper ends of the robot. Since the human hand can be opened wider than the robot gripper, we map the distance between both gripper ends to a range between 0 and 14 cm.

**FIGURE 8:**
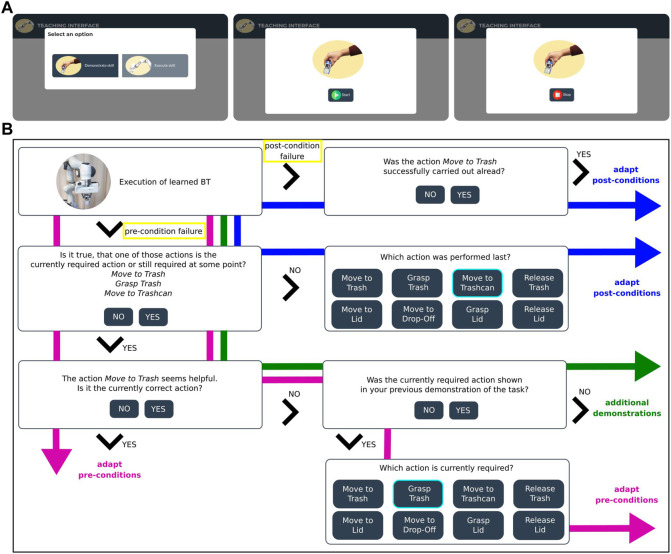
**(A)** Overview of the web-interface for demonstrating and recording a new task. First, the user selects the button “Demonstrate skill”. The recording is then started and stopped by pressing the “Start” and “Stop” button. **(B)** Webinterface dialogue for different failure cases (yellow) and corresponding answer possibilities. If a post-condition fails, the user can confirm to adapt the failed condition (blue). In case of a pre-condition failure, the user provides further information about the currently required action in order to adapt its pre-conditions (magenta). If the robot is currently trying to achieve an action that has already been executed, its post-conditions have to be adapted (blue). In case the currently required action was not shown in the demonstrations, additional demonstrations are needed (green).

In written instructions, the participants were asked to watch the robot while it performs the learned task and provide guidance if the robot asks for help via the web-interface. Based on this input, the initial BT and learned conditions were updated as described in Section 3.3. After the robot successfully solved the task once with the help of the user, the robot executed the updated BT a second time. Since the BT is updated using the user’s input, we expect fewer failure cases and, therefore, fewer requests for help the second time. The web-interface dialogue for different failure cases is schematically shown in Figure 8B.

In Table 4, all adjusted pre- and post-conditions for the first and second task execution are summarized for all ten successfully built BTs from human demonstrations. For this analysis, we exclude the cases where the participants continued with a fallback BT since the same structure and condition ranges of this fallback BT lead to similar failure cases during robot execution. On average, 4.67 pre-conditions and 1.89 post-conditions were changed during the first robot execution per participant. The required user input for the execution of the already adjusted BT was less, with an average of 1.78 pre-conditions and 0.11 post-conditions adjusted according to the user input. The number of required adjustments can vary, among other things, based on the learned structure of the BT. Reasons for failures during the execution of the initial BT in the first robot execution run are mostly differences between the implementation of the robotic actions and how the users demonstrated them. In the first experiment run, the pre-condition of the action *Move-to-Trash* regarding the feature *dis_hand_trash* failed seven out of ten times. The reason lies in the implementation of the robot action *Move-to-Trash*, since the robot first moves to a pre-grasp position above the trash, and thereby the distance between the gripper and trash exceeds the upper limit of the feature range shown in the human demonstrations. A different implementation of the robotic action could avoid this failure. However, because of the proposed interactive approach, such failure cases can still be solved during execution. The lower number of failure cases during the execution of the already adjusted BT shows that the BT is improved through the interaction with the user. It has to be mentioned that the comparably high number of failures for action *Move-to-Trash* regarding the feature *dis_xy_trash_trashcan* during the second run occurred 5 out of 6 times during the interaction with one particular user. This user confused the actions *Move-to-Trash* and *Move-to-Trashcan* which resulted in sub-optimal input and a repeated failure of this pre-condition until the user realized the mistake. A more detailed explanation of all actions in the web-interface in the form of an additional info button could improve the interaction and was also suggested in some of the user’s comments in the subsequent user experience questionnaire. Other reasons for failures during the second run of the improved BT can be inaccuracies in the object tracking that cause a feature to exceed the learned and already improved feature ranges. For all 20 participants, both the first and second robot execution of the BT ended with successful completion of the task with the help of the user input.

**TABLE 4:** Analysis of pre- and post-condition changes during robot execution for different actions and features for the first and second robot execution.

Pre-condition	Move to Trash		Grasp Trash		Grasp trashcan		Release Trash		Goal	
	1st	2nd	1st	2nd	1st	2nd	1st	2nd	1st	2nd
dis_xy_trashcan_lid	0	0	0	0	0	0	0	0	0	0
dis_xy_trash_trashcan	1	6	1	2	4	2	0	0	0	0
dis_z_trash_trashcan	0	0	0	0	9	0	1	0	0	0
dis_hand_trash	7	0	0	0	0	0	0	0	5	3
dis_fingers	0	0	5	0	0	0	0	0	0	1
dis_hand_lid	0	0	1	1	1	0	11	0	0	1
**Total**	**8**	**6**	**7**	**3**	**14**	**2**	**12**	**0**	**5**	**5**

**Post-condition**	Move to Trash		Grasp Trash		Grasp Trashcan		Release Trash		Goal	
	1st	2nd	1st	2nd	1st	2nd	1st	2nd	1st	2nd
dis_xy_trashcan_lid	0	0	0	0	0	0	0	0	0	0
dis_xy_trash_trashcan	0	0	0	0	2	1	0	0	0	0
dis_z_trash_trashcan	0	0	0	0	0	0	5	0	0	0
dis_hand_trash	4	0	0	0	0	0	0	0	0	0
dis_fingers	1	0	7	0	0	0	0	0	0	0
dis_hand_lid	0	0	0	0	0	0	0	0	0	0
**Total**	**5**	**0**	**7**	**0**	**2**	**1**	**5**	**0**	**0**	**0**

#### 4.3.4 User experience

**FIGURE 9:**
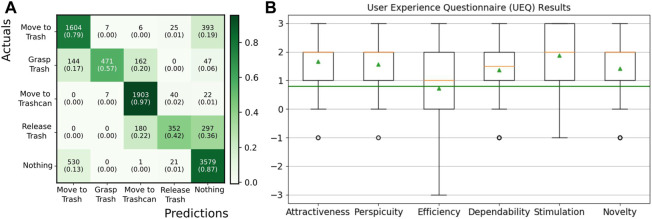
**(A)** Confusion matrix showing the predicted high-level actions compared to the manually labeled ground truth actions. The absolute number of frames, as well as the normalized value is reported in brackets below. **(B)** Results for the six constructs of the User Experience Questionnaire (UEQ) over 17 participants. The median is shown in orange and the green triangles show the mean. Values above 0.8 (green line) are considered a positive evaluation.

The results of the User Experience Questionnaire (UEQ) (Schrepp et al., 2017) are shown in Figure 9. We excluded the answers of three participants since their answers showed a big difference between the evaluation of different items of the same scale. This is considered a problematic data pattern in the UEQ and hints at random or not serious answers. For the remaining 17 participants, all six constructs of the UEQ questionnaire show a median (orange) of above 0.8, which is considered a positive evaluation. Here, the range of the scale is between −3 (horribly bad) and +3 (extremely good). In particular, the constructs “Attractiveness” (Mean: 1.70, Mdn: 2.0), “Perspicuity” (Mean: 1.61, Mdn: 2.0), “Dependability” (Mean: 1.40, Mdn: 1.5), “Stimulation” (Mean: 1.88, Mdn: 2.0), and “Novelty” (Mean: 1.50, Mdn: 2.0) were rated positively. The only item that was evaluated with a value below 0.8 on average is the item “slow/fast” with a value of −0.6, which represents a more or less neutral evaluation. This results in lower values for the corresponding construct “Efficiency” (Mean: 0.83, Mdn: 1.0). One reason could be the design of the web-interface that required the user to press “Trash Disposal” again in the web-interface after every interaction and corresponding refinement of the BT to continue the robot execution. Three participants suggested automatically continuing the task in their comments and removing this step that was considered unnecessary.

#### 4.3.5 Reactivity of learned behavior trees

**FIGURE 10:**
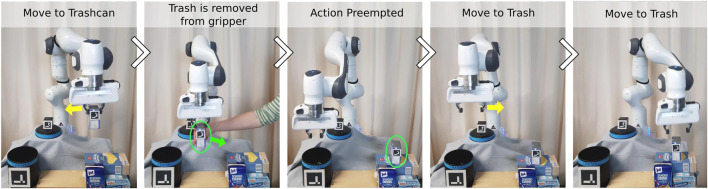
Example showcasing the reactivity of the learned BT. The robot picked up the trash and moves towards the trashcan. Inbetween the action *Move-to-Trash*, the trash is removed from the gripper and placed somewhere else (highlighted in green). The action is preempted and the robot switches again to the action *Move-to-Trash*.

Reactivity is one of the main advantages of Behavior Trees. Our approach of interactively learning a Behavior Tree with continuous pre- and post-conditions from human demonstrations preserves this reactivity. This requires all actions to be implemented as reactive action nodes, so that the robot can react to external changes during execution. One example of is shown in Figure 10. The robot first moves to the trash, grasps it, and starts to move toward the trashcan. In between the action *Move-to-Trashcan*, the trash is removed from the robot’s gripper and placed again on the starting position. Since the pre-conditions of the action *Move-to-Trashcan* are not fulfilled anymore when the trash is removed from the gripper, the action *Move-to-Trashcan* is preempted. The next tick of the BT triggers the action *Move-to-Trash* and the robot moves toward the trash again. This example showcases the reactivity of the learned BT. If an action fails during execution, the robot can adapt to the new situation and still successfully execute the task. Examples for such failure cases for the given trash disposal task could be that the robot fails at grasping the trash or drops the trash while moving toward the trashcan.

#### 4.3.6 Including additional human demonstrations

**FIGURE 11:**
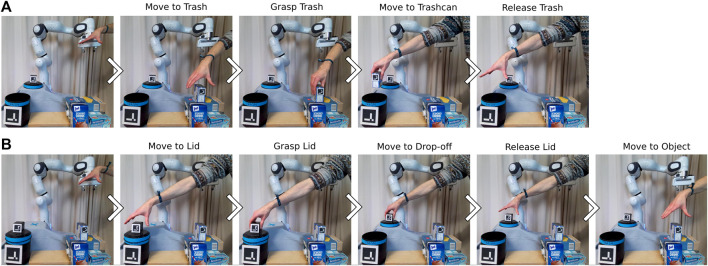
**(A)** Initial human demonstrations of the trash disposal task with the lid already set aside at the beginning. **(B)** In order to be able to execute the task if the lid is on top of the trashcan, the user has to show additional demonstrations showing how to set aside the lid before the *Move_to_Trash* action.

In addition to the interactive refinement of action conditions, our approach allows to include additional demonstrations in the initially learned BT, as described in Section 3.3.3. This way, it is possible to include new actions or action sequences if an unseen situation requires so. In our study on how non-expert users demonstrate the task (Section 4.2) we saw that most users only demonstrate how to dispose of the trash if the lid of the trashcan is already set aside. A BT learned from such imperfect demonstrations that can not solve the task successfully if the lid is placed on top of the trashcan. If the user wants to teach the robot how to first set aside the lid, it is necessary to show additional demonstrations of the required actions and include them in the initial BT. It would be possible to teach the robot a new BT from scratch by demonstrating the whole task starting with the lid on top of the trashcan three times. However, by just demonstrating the part of the task unknown to the robot and exploiting the already learned BT the user effort can be kept low. First, an initial BT is built from three human demonstrations starting with the lid already set aside, as shown in Figure 11A. If the robot executes this BT and the lid is on top of the trashcan, the pre-condition of the action *Move-to-Trash* regarding the feature *dis_trashcan_lid* fails. Since the currently required action was not shown in the previous task demonstrations, the user has to provide new demonstrations until the end of the action corresponding to the failed pre-condition (here *Move-to-Trash*), as shown in Figure 11B. This action must be included in the demonstrations to ease the transition between the new actions and the first action of the initial BT. A subtree is then added to the initial BT based on the additional demonstrations as described in Section 3.3.3. In Figure 11, we exemplary show that a BT learned from human demonstrations (Figure 6A) can be successfully extended using additional human demonstrations. The resulting BT is illustrated in Figure 6B. The corresponding subtrees of the initially learned BT are highlighted in yellow and green. The subtree corresponding to the additional demonstrations is highlighted in magenta. For better readability, some actions and subtrees are collapsed.

## 5 Conclusion

In this paper, we introduced ILBERT, a new framework to interactively learn a BT from human demonstrations. In contrast to related approaches, we directly learn a BT from only a few RGB-D video recordings of human task demonstrations and automatically extract a set of continuous pre- and post-conditions for action executions from visual features. In a study on how non-expert users demonstrate tasks to a robot, we identified three main causes for failures when learning a BT from a few human demonstrations. We automatically detect and resolve these failure cases at runtime by requesting interactive help from the user via a web-interface and adapting the BT and corresponding conditions based on the user input.We evaluated the resulting system on a robotic trash disposal task with 20 subjects. While the automatic condition computation, BT building, and interactive refinement showed good results, the action classifier used for experiments in this paper should be replaced by a more robust approach in the future. Evaluation of the UEQ revealed an overall high level of user satisfaction with the developed system.

### 5.1 Limitations

We believe that our approach of interactively learning robot behaviors in form of Behavior Trees from potentially imperfect human demonstrations offers a user-friendly way to teach a robot new skills. However, there are still several limitations to the proposed work. The approach was evaluated on a rather simple pick-and-place task and it would be interesting to see how the approach could extend to more complex task scenarios. The BT-based framework allows arbitrary actions to be implemented as action nodes. However, a reliable object tracking and action recognition is required in order to be able to compute meaningful conditions and build an executable Behavior Tree. In addition, task-relevant features have to be pre-defined. While we propose a new method to extract continuous pre- and post-conditions directly from human demonstrations, the method requires a number of task-dependent hyper-parameters.

### 5.2 Future work

For future work, we consider it interesting to extend the proposed framework to learn from multimodal human demonstrations and also offer multimodal interaction channels such as speech and gestures.Moreover, we want to explore alternative approaches for automatic pre- and post-condition extraction from human demonstrations across different task settings. Abdo et al. (2013) cluster feature values at the beginning and end of an action where each cluster represents a different way how an action was demonstrated. They introduce a variance measure based on these clusters in order to identify relevant conditions of an action. A similar approach could be used to improve the current condition computation, as well as to determine relevant action conditions out of a larger set of features as the set of task-relevant features.We also want to conduct a further study on how non-experts can be better guided to provide meaningful additional demonstrations in case of failures due to unseen situations and whether they can learn over time how to provide a more complete set of few initial demonstrations for the robot to learn from. Another interesting future direction is to use interactive human input within the proposed framework not only to resolve BT failures at execution time but to additionally include user feedback to refine the action classifier in a semi-supervised manner (Gassen et al., 2023; Rangnekar et al., 2023) or for interactive object detection (Lombardi et al., 2022).

## Data Availability

The raw data supporting the conclusion of this article will be made available by the authors, without undue reservation.
